# One Health, One Disease

**Published:** 1877-10

**Authors:** P. H. Hayes


### ONE HEALTH, ONE DISEASE.

P. H. HAYES, M. D.

**Second Article.**

There is a grandly mysterious force in that system of organs which converts food into blood. We eat bread and butter, beef-steak, what not, and we say it is upon these that we live. In a more strict sense we live upon the blood which our digestive or blood-making organs make from the food we consume. It is the blood that tells, and through it life is renewed, or disease makes its entrance into the body. First in the order of life, and first in importance then is a knowledge of the blood-making organs. In them actually begins the *one health*, or the *one disease*. Of all the conditions by which the one health shall be forever maintained, and the one disease forever avoided, no one is so elementary as the quality of our food.

In the treatment of chronic diseases, I am continually in the habit of writing the following *pro*scription as to diet: Must not eat pork; salted or cured meats; uncooked vegetables, except celery and tomatoes, nor uncooked fruits, nor pastry, hot or fresh bread, dry toast, crackers, rich cake, cheese, spices, nor anything *fried*.

I exclude pork because it is difficult of digestion. In Dr. Beaumont's experiments on St. Martin he found it required more than five hours for complete digestion, and this in a robust man in the prime of life. Yet this very quality makes it the best of food for soldiers, sailors, miners, hunters and lumbermen—all who work hard in the open air. Said an old Adirondack hunter, out with your venison and trout, give me pork. Pork sticks by the rib. I can work on pork. Pork requires a vigorous out-door life for its digestion. It is certainly injurious, as a rule, to old people, to women and children, to professional men and to all invalids.

I exclude salted and cured meats because by this process the fibre of the meat is hardened and rendered very difficult of digestion, and moreover the ingestion of so much salt, or other curing material, imparts a feverish condition to the blood, excites unnatural thirst and impairs nutrition.

Uncooked vegetables of every sort, except celery and tomatoes, are difficult of digestion and can be eaten by those only who can master a pork diet.

I started out in treating chronic diseases with the conviction that fresh ripe fruit was good for all, and to be eaten freely at meals and by the most vigorous at all times, not only with impunity but with positive advantage. I now exclude absolutely all fruit between meals, and in all cases of asthma, sick-headache, tic doloreux, epilepsy and the whole family of neuroses and skin diseases, I forbid fruit utterly except it be *stewed*, and *well stewed* too, and then only to be taken morning and at noon.

Nothing in this country is so sure to spoil the digestion of the dinner as the everlasting pastry at the end of it. Heavy with pork grease, solid and sodden in its under crust, it sets like lead upon a weak stomach, and with all persons you may see how nature toils with it by the red face after dinner, and the dullness of mind, apathy and bodily lassitude which follow. Many school children suffer in the afternoon from indigestion and inaptitude to study, brought on by pie, and especially mince pie, for lunch. The questions in algebra won't come out right, the States won't bound, the rivers won't run the right way, "can't think of a thing to write for composition," the brains are down in the stomach to work on that pie.

Moreover, pastry is constipating. Hot and fresh bread are constipating. Dry toast is constipating. Crackers are constipating and are often made of superfine flour, and they *can* be made of flour which is too stale for good bread. Cheese is theoretically good food, but too difficult of digestion and too constipating for any but the hardy out-of-door laborer. If we sum up right here these few things, pastry, hot bread, dry toast, crackers, cake, cheese and spices, we shall find every one of them will produce constipation, and often are at the bottom of that very troublesome and painful disease, the piles. No affection is more common, yet few have in its trail more evils than habitual constipation. Not to mention piles, we find headache, loss of appetite, a foul tongue, bad breath, langour and depression of spirits are abundantly common.

Of all the methods of cooking food which are really detestable, that of frying is most detestable of all. The meat or other material thus cooked is commonly charred by the intense heat of the fat and this may be in the case of meat though not blackened at all. Moreover the fats are decomposed giving rise to new empyreumatic compounds more or less acrid, and impossible of digestion. When we recall the pies and doughnuts, the fried pork swimming in hot fat, the hot saleratus biscuit and strong coffee which were the very staples of life a generation ago, we do not wonder that our present national disease is dyspepsia. We do not wonder that every doctor says "*bilious*" to his patient, and that the very look and expression of fine health is rare, even among the young.

I stated almost in the outset my usual *pro*scription as to diet, and I have followed in some detail with reasons for the several proscriptions. I am often greatly amused when I read off the list of things forbidden, for if my patient belongs to the old school of livers, he will stare and hold his breath, evidently wondering on what he is to live under the new regime. And so I hasten on to relieve his terrible suspense by saying:

May eat bread and butter, stewed or canned or baked fruits, except at supper, bread and butter with honey if it agrees, bread and milk, rare eggs, fresh fish, game, oysters and poultry. Also fresh beef and lamb, roast, boiled or broiled. Also baked potatoes, thoroughly boiled onions, tomatoes baked and celery.

I need not say a word as to why bread should be the *alpha* in the alphabet of things to eat. As to butter it is not so obvious, and there is a strong anti-butter party in dietetics. There is among all nations an instinctive desire for oleaginous food. In bible times it is very evident that ideas of plenty and luxury were associated with "*butter and honey.*" "Oil that maketh his face to shine." And Canaan was represented as "a land of oil olive." It is well known what quantities of train oil, whale blubber and even tallow candles the Greenlanders and Esquimaux will devour if they can get them. Even the Hindoo, in the torrid zone, will spend all the "*pice*" he can get for the clarified butter of the country; and "as good as Ghee!" is his proverb. Good fresh butter is certainly very digestible and most readily available for the indispensable wants of nature. Indispensable, indeed, for *phthisis* and many other wasting and nervous diseases. Certain forms of neuralgia and rheumatism, and some skin diseases have their whole gist and essence in the indigestion of fats. And it is right here, and for this reason, that cod liver oil, an already digested fat, has made such really brilliant cures.

I exclude fruit in any form at supper partly because it is apt to cause flatulence over night and thus to cause dreaming and troubled sleep, and partly because it adds one item too many for the very light supper which should be taken.

Let no one forget while reading this article that all its teachings are for those of weak digestion. For this class of persons milk is one of the best of all foods, because, being easy of digestion, it is not long detained in the stomach and it does not require the vigorous activity of this organ which is required by beef or lamb. The richer the milk is in the element of cream the more nutritious and digestible it is for the adult human stomach.

Milk is designed by nature only for the infant in a rapid condition of *growth* and at a time of life when *work* or active *force* is not required. For this reason milk is far from being as perfect food for an adult at work either with muscle or brain, yet for this very reason good for invalids, of whom work is impossible.

Rare eggs, that is eggs cooked in water at 210 degrees for five minutes, are most excellent and most nutritious food for all persons. Fresh fish broiled and not dressed with butter, but barely moistened on the surface with cream, is a great luxury for sick or well. Game and poultry boiled or broiled are very easy of digestion. For invalids who are obliged to attend to some business, or who can take considerable exercise in the open air daily, beef or lamb should form the center of at least one meal in the day. Either of these meats roasted and sliced cold for supper form, with bread and butter, a most sensible and excellent repast for this hour of the day.

The most perfect mode of cooking potatoes is baking or roasting. A good potato is then dry and mealy, and is light and remarkably easy of digestion. Thoroughly boiled onions are most excellent food, a real tonic to stomach and bowels, and to the kidneys also. For obvious reasons it is difficult to persuade most invalids to eat them. Tomatoes are excellent food. If baked, and then turned out of the skin and seasoned lightly with sugar or salt, they are more easily digested than otherwise.

I say to my patients, eat but one kind of animal food at a meal. Milk is certainly incompatible with all kinds of meat and fish, though it is not so with eggs, and for the sake of simplicity in diet it is better even here to obey the rule above given. To many invalids I allow soft pickles but only with fish or meats.

Coffee and tea certainly weaken digestion, *directly* by keeping up a partial paralysis of the organic and pneumogastric nerves, and *in*directly by introducing too much liquid into the stomach. I try to explain to my patients that the habit of drinking anything at meals is a bad one, as thereby the food is washed down too quickly into the stomach. In truth a considerable amount of digestion takes place in the mouth if the food is held there for a time and thoroughly masticated, without drinking. But when hurried on by drinking, the food is not ready for the stomach, nor the stomach ready for it, and discomfort and distress ultimately follow this practice. If coffee and tea must be taken they should be *sipped* not *drank*, and the habit of using and being perfectly satisfied with a very little will soon be established.

